# A gene expression based predictor for high risk myeloma treated with intensive therapy and autologous stem cell rescue

**DOI:** 10.3109/10428194.2014.911863

**Published:** 2014-08-19

**Authors:** Ping Wu, Brian A. Walker, Annemiek Broyl, Martin Kaiser, David C. Johnson, Rowan Kuiper, Mark van Duin, Walter M. Gregory, Faith E. Davies, Daniel Brewer, Dirk Hose, Pieter Sonneveld, Gareth J. Morgan

**Affiliations:** 1 Section of Haemato-Oncology; 2 Department of Hematology, Erasmus University Medical Center, Rotterdam, The Netherlands; 3 Clinical Trials Research Unit, University of Leeds, Leeds, UK; 4 Prostate Cancer Genome Team, Institute of Cancer Research, Sutton, Surrey, UK; 5 Medizinische Klinik V, Universitaetsklinikum Heidelberg, Heidelberg, Germany

**Keywords:** Myeloma, gene expression profiling, risk, predictor

## Abstract

Myeloma is characterized by a highly variable clinical outcome. Despite the effectiveness of high-dose therapy, 15% of patients relapse within 1 year. We show that these cases also have a significantly shorter post-relapse survival compared to the others (median 14.9 months vs. 40 months, *p* = 8.03 × 10^− 14^). There are no effective approaches to define this potentially distinct biological group such that treatment could be altered. In this work a series of uniformly treated patients with myeloma were used to develop a gene expression profiling (GEP)-based signature to identify this high risk clinical behavior. Gene enrichment analyses applied to the top differentially expressed genes showed a significant enrichment of epigenetic regulators as well as “stem cell” myeloma genes. A derived 17-gene signature effectively identifies patients at high risk of early relapse as well as impaired overall survival. Integrative genomic analyses showed that epigenetic mechanisms may play an important role on transcription of these genes.

## Introduction

Multiple myeloma (MM) is a malignancy of plasma cells which accumulate in the bone marrow, causing clinical symptoms as a consequence of myelosuppression, osteolysis and the production of monoclonal protein [1]. Although the advent of new agents during the last decade has made therapies in MM more diverse, high-dose therapy (HDT) followed by autologous stem cell transplant (ASCT) remains a standard treatment for newly diagnosed patients with myeloma who are considered to be able to tolerate the procedure. Compared to prior standard treatments it has been shown to increase the median overall survival (OS) by a year to 4–5 years [2,3]. The incorporation of novel agents as part of induction and maintenance has improved outcomes further [4–6]. However, virtually all patients with transplant eventually relapse, and the duration of remission is highly variable, ranging from a few months to more than 10 years. The difference in outcome is thought to be mediated via tumor acquired genetic differences [7]. A diagnostic test able to identify patients at high risk of early relapse based on such genetic differences would be of great clinical utility, as it would allow clinicians to design and implement trials investigating new therapeutic strategies in this high risk subset. Any test used in such a setting should have high specificity for the correct identification of high risk behavior as well as have good sensitivity to be able to identify such cases.

Until now a number of approaches have been used to determine risk status. The initial approach used β_2_- microglobulin (β2M) level, which was subsequently incorporated into the international staging system (ISS) [8]. While the ISS is generally applicable, it works best for classifying populations entered into clinical trials and lacks biological relevance. Genetic variables associated with poor outcome have been identified, and fluorescence *in situ* hybridization (FISH) studies have identified the presence of del(17p13), gain(1q21) and del(1p) as well as adverse translocations t(4;14), t(14;16) and t(14;20) as high risk factors [9–13]. However, despite the fact that this approach can identify distinct biological groups, the sensitivity and specificity for identifying high risk behavior are too poor to be used as a diagnostic tool, and developing alternative approaches is a priority to reliably identify patients at high risk.

Gene expression profiling (GEP) offers a potential solution to identify high risk behavior, and gene signatures based on global GEP of presenting samples have been explored [14–16]. However, they still have a number of drawbacks, including a failure to take account of known cytogenetic subgroups with distinct clinical behaviors. In order to develop a novel risk predictor for patients treated with HDT, we have utilized a series of uniformly treated patients with myeloma and driven the analysis using early relapse as the endpoint to develop a signature able to identify high risk cases.

## Materials and methods

### Patients

The Medical Research Council (MRC) Myeloma IX trial (ISRCTN68454111) enrolled 1960 patients with newly diagnosed symptomatic myeloma who were allocated to two main treatment pathways, intensive (*n* = 1111) or non-intensive (*n* = 849), at the discretion of the treating physician, taking account of age and performance status [4]. For the purpose of reliably defining subjects with early relapse following the HDT procedure, cases used in this study were all based on per protocol rather than intention to treat. Of the 1111 patients in the intensive arm, 747 cases who actually received HDT were used for subsequent analyses [Supplementary Figure 1(A) available online at http://informahealthcare.com/doi/abs/10.3109/10428194.2014.911863]. Early/late relapse subgroups were defined by calculating the duration from the time of HDT to subsequent relapse.

Gene expression profiles from CD138-selected bone marrow plasma cells (to a purity of more than 90%) of 261 patients from Myeloma IX (GSE15695) were collected as previously described [17]. In addition, two other similar publicly available gene expression sets were used in this study for development of a GEP-based predictor. Ninety-seven evaluable cases from Myeloma IX and 82 evaluable cases from the Hemato-Oncology Foundation for Adults in the Netherlands (HOVON)-65 trial (GSE19784; ISRCTN64455289), 18 being treated with single HDT, were combined to form a training set of 179 samples, and batch effect was removed using Bioconductor package Combat 19. In general, cases who died from causes other than progressive myeloma (mostly other cancers, heart disease, stroke and infection) within 1 year post-HDT were excluded, as the relapse status at 1 year post-HDT could not be assessed. As there was partial overlap of samples between HOVON-65 and GMMG-HD4 datasets, the subjects present in both datasets were excluded from the training set to ensure the independence of the test set. Following the same selection criteria, 155 patients from the German-Speaking Myeloma Multicenter Group (GMMG)-HD3/HD4 trial (E-MTAB-362; ISRCTN06413384) [20], of whom 56% were treated with double HDT, were used as a validation set for the GEP-based predictor. A summary of samples used in this study, therapy schedule and analysis flow is outlined in Supplementary Figure 1(B) available online at http://informahealthcare.com/doi/abs/10.3109/10428194.2014.911863. The detailed designs of these trials have been reported previously [4,6,21]. A further independent dataset (GSE24080) was used to validate its effect on PFS and OS.

Genes identified as being differentially expressed were correlated with matching SNP-based mapping (*n* = 99) and DNA methylation profiling (*n* = 118) data from the Myeloma IX trial to explore possible mechanisms underlying deregulation.

### Bioinformatics and statistical analysis

GEP of all samples from the training and test sets was carried out on the Affymetrix Human Genome U133 Plus 2.0 platform, and gene expression signals were quantified using robust multi-array average (RMA) normalization. All analyses were performed in R 2.10.1 and Bioconductor. Differentially expressed genes between patient groups were selected using significance analysis of microarray (SAM) (Bioconductor package *samr*) with a 1000-permutation adjustment. The LASSO algorithm (Bioconductor package *glmnet*) was used to further refine the selection to a subset of non-correlated genes with strongest discriminative power for early relapse. The selected genes were fitted in a logistic regression model to obtain an optimal model, and a risk score (z) for early relapse was calculated by a linear combination of the expression levels of the 17 selected genes at presentation, weighted by their estimated regression coefficients; subsequently the probability for early relapse could be calculated accordingly (Table III). The predictive power (sensitivity/specificity) of a model was tested using a receiver operating characteristic (ROC) method and the corresponding area under the curve (AUC) was calculated, which can be interpreted as the chance of getting the prediction correct.

The associations between early and late relapse groups and various clinical parameters were investigated using Fisher's exact test for categorical parameters and Wilcoxon test for continuous variables. Any parameters statistically associated with early relapse were combined in a multivariate logistic regression model to test their independence. Performance of the predictive models was compared using the likelihood-ratio test (R package *anova.glm*). Either Wilcoxon or Kruskal–Wallis test was used to correlate the gene expression level and single nucleotide polymorphism (SNP)-mapping as well as DNA methylation data where appropriate. As the impact of DNA methylation on gene expression is thought to result from a discrete methylation pattern (hyper- or hypo-methylation), we used the unsupervised *k*-means method to define the high/low methylation groups for each gene. The binary distribution of promoter methylation of the genes was visualized by Kernal density plot. The distribution of OS and progression-free survival (PFS) between risk groups was estimated using the Kaplan– Meier method (log-rank test). The performance of the derived high-risk gene signature (REL-17) was compared with another gene signature using multivariate logistic/Cox regression analysis. Pathway analyses were performed using GeneGo's MetaCore (www.genego.com).

## Results

### Defining a patient group with high risk clinical behavior following HDT

**Figure 1.**
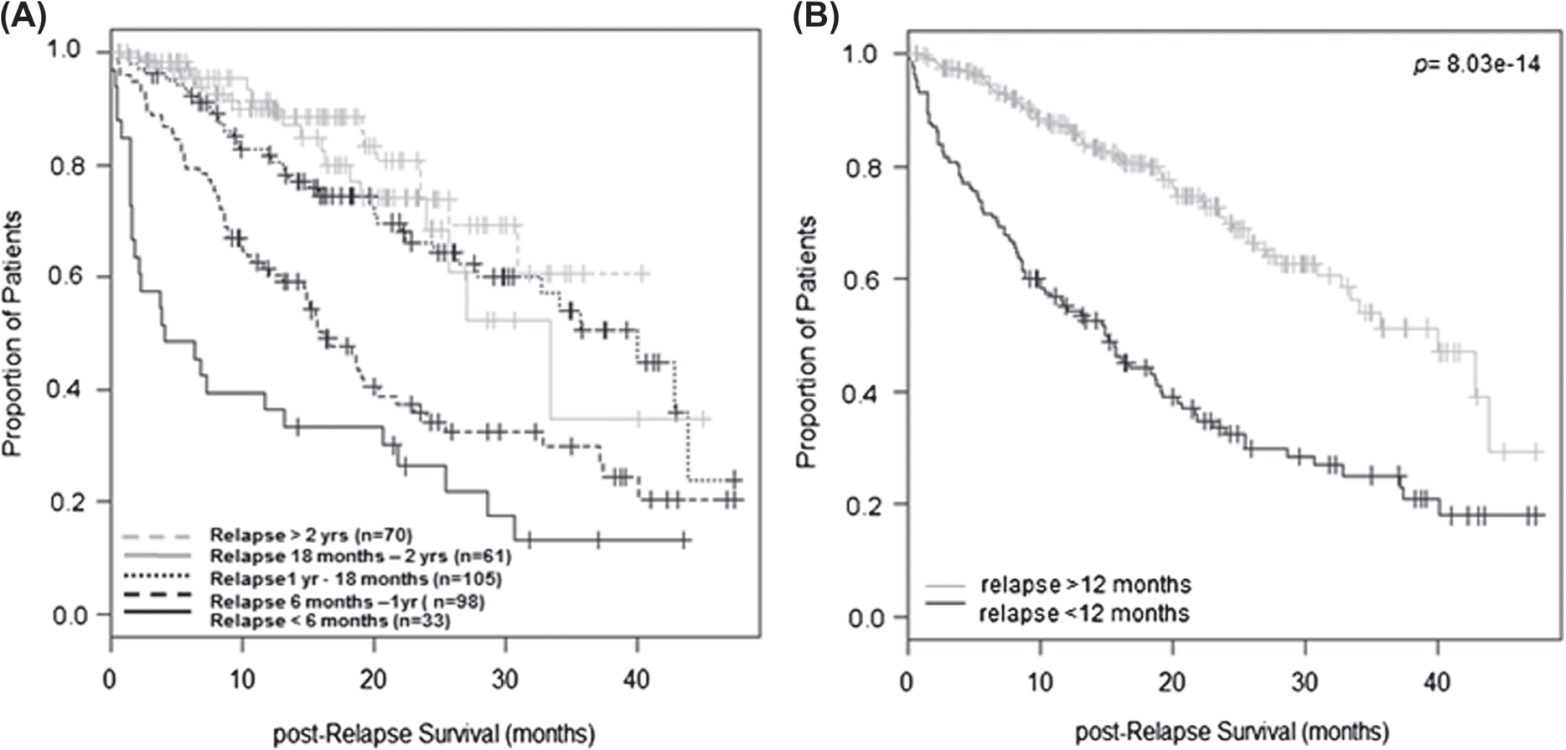
Impact of remission duration on post-relapse survival according to data from Myeloma IX. (A) Analyses on 423 relapsed cases show a cut-off effect of relapsing within 1 year on post-relapse survival: median 4.1 months (< 6 months), 16.1 months (6 months–1 year), 40 months (1 year–18 months), 33.4 months (18 months–2 years) and not reached (> 2 years). (B) When combined, patients who relapsed within 1 year post-HDT had median post-relapse survival of 14.9 months in contrast to 40 months with those who relapsed at later point (log-rank test *p* = 8.03 × 10^− 14^).

In order to identify a group of patients who have poor outcome post-HDT, 423 out of the 747 patients from Myeloma IX who relapsed after HDT were split into subgroups based on time to progression. The results showed that patients who relapsed within 6 months and those who relapsed between 6 months and 1 year had significantly shorter post-relapse survival compared to the others [Figure 1(A)]. When combined, these cases had a median post-relapse survival of 14.9 months, in contrast to 40 months for those who relapsed after 1 year [Figure 1(B), log-rank test *p* = 8.03 × 10^− 14^], suggesting that they may represent two biologically distinct groups.

### Clinical and FISH parameters associated with high risk behavior

Among the 747 patients treated with upfront HDT, relapse status at 1 year post-HDT was available in 718 cases, among whom 18.2% (131 out of 718) relapsed within 1 year post-HDT. In order to compare the associated clinical and FISH parameters of this group with the rest of cases (*n* = 587), univariate logistic regression analyses were performed on parameters including gender, age, World Health Organization (WHO) performance status, hemoglobin (Hb), platelets (Plt), albumin (Alb), β_2_M, creatinine (Cr), calcium (Ca), lactate dehydrogenase (LDH) and C-reactive protein (CRP), paraprotein type (immunoglobulin A [IgA] vs. non-IgA), light chain type, duration from diagnosis to HDT, type of induction therapy, thalidomide maintenance, adverse immunoglobulin heavy chain (IgH) translocations [including t(4;14), t(14;16) and t(14;20)], del(17p), gain(1q) and del(1p). The results show that only low Hb, low Plt, presence of adverse IgH translocations and gain(1q) at presentation were statistically associated with early relapse (*p* < 0.05). When tested together in a multivariate logistic regression model, only adverse IgH translocations and gain(1q) retained statistical significance (Table I). A predictor for early relapse was developed based on these two FISH abnormalities, but this only had modest predictive power (AUC 0.72 in the whole Myeloma IX dataset and 0.69 in both expression training and test datasets, Supplementary Figure 2 available online at http://informahealthcare.com/doi/abs/10.3109/10428194.2014.911863), and therefore was discarded.

**Table I.** Associations of clinical and FISH parameters with early relapse were evaluated in 718 patients from Myeloma IX trial∗.

	Univariate		Multivariate	
Predictive factors at diagnosis	Odds ratio	*p*-Value	Odds ratio	*p*-Value
Hb < 10 (g/dL)	2.2	< 0.001	0.6	0.43
Plt < 133 (10^9^/L)	2.4	0.007	1.1	0.74
Adverse IgH translocations	6.7	< 0.001	4.4	< 0.001
Gain(1q)	4.1	< 0.001	2.8	0.001

∗ Hb, Plt, adverse IgH translocations [including t(4;14), t(14;16) and t(14;20)] and gain(1q) were significantly associated with early relapse in univariate analyses; however, only adverse IgH translocations and gain(1q) remained significant in a multivariate analysis which was performed in 348 patients with completed dataset on these variables.

### Development of a GEP-based predictor for early relapse

We investigated whether a GEP-derived predictor could improve or outperform the FISH-based predictor. Among the 179 patients in the training set, 22.3% (40 patients) relapsed within 1 year post-HDT, which is comparable to the complete dataset. The GEP of these two groups of patients was compared using significance analysis of microarray (SAM) [22], and 207 genes were identified as being differentially expressed at 5% false discovery rate (FDR), among which 173 were up-regulated in early relapsed patients and 34 were down-regulated (Supplementary Table I available online at http://informahealthcare.com/doi/abs/10.3109/10428194.2014.911863). Gene enrichment analysis by chromosome location showed that there was a significant overrepresentation of genes from chromosomes 1 and X (*p* < 0.001, Supplementary Table II available online at http://informahealthcare.com/doi/abs/10.3109/10428194.2014.911863). Fifteen of the 173 up-regulated genes span a region corresponding to 1q21-q23, which might reflect the poor outcome associated with gain(1q) by FISH. Notably *WHSC1, FGFR3* and *MAF* were among the top differentially expressed genes, known to be deregulated via t(4;14) translocation and t(14;16), respectively.

In order to develop a robust yet manageable GEP-based predictor for early relapse, we repeated the analysis with a more stringent FDR of 0% and obtained 37 differentially expressed genes, among which 30 were up-regulated in early relapsed patients while seven were down-regulated (Table II). The 37 genes still showed marked overrepresentation of genes from chromosome X (*p* = 0.0001), while the overrepresentation of genes from chromosome 1 was no longer seen. Among these 37 genes, pathway analyses identified significant enrichment of epigenetic regulators, including genes involved in histone modification (*WHSC1, HIST1H4H*) as well as other chromatin modificators *NAP1L3* and *HMGN5 (p* < 0.05, adjusted for Benjamini–Hochberg multiple testing). Notably, six of the top deregulated genes (*NUDT11, PKP2, ROBO1, AGAP1, NAP1L3* and *EPDR1*) were recently identified as “stem cell” genes in myeloma [23].

**Table II.** Top 37 deregulated genes at FDR 0, among which 30 were up-regulated (A) while seven were down-regulated (B) in early relapse cases in training set∗.

Gene ID	Gene symbol	Score (d)	Fold change	Cytoband	GO-term/description
A					
** 210546_x_at**	**CTAG1A /// CTAG1B**	**− 4.92**	**0.54**	**Xq28**	**Cancer testis antigen 1**
** 223253_at**	**EPDR1**	**− 4.71**	**0.46**	**7p14.1**	**Cell-matrix adhesion**
** **215733_x_at	CTAG2	− 4.69	0.48	Xq28	Cancer testis antigen 2
** 219895_at**	**FAM70A**	**− 4.17**	**0.31**	**Xq24**	**—**
** 207717_s_at**	**PKP2**	**− 4.09**	**0.40**	**12p11**	**Cell adhesion**
** **207307_at	HTR2C	− 4.02	0.51	Xq24	cGMP biosynthetic process, signal transduction, response to drug
** **217963_s_at	NGFRAP1	− 3.82	0.41	Xq22.2	Apoptosis
** **211596_s_at	LRIG1	− 3.80	0.54	3p14	DNA replication, DNA repair
** **201037_at	PFKP	− 3.75	0.66	10p15.3-p15.2	Protein homotetramerization
** 204379_s_at**	**FGFR3**	**− 3.75**	**0.22**	**4p16.3**	**Signal transduction, cell proliferation, cell differentiation**
** **204749_at	NAP1L3	− 3.68	0.58	Xq21.3-q22	Nucleosome assembly
** **204066_s_at	AGAP1	− 3.66	0.51	2q37	Signal transduction, protein transport
** **217975_at	WBP5	− 3.64	0.48	Xq22.2	Mediating protein–protein interactions
** 213194_at**	**ROBO1**	**− 3.57**	**0.42**	**3p12**	**Cell migration/adhesion, cell differentiation, activation of caspase activity**
** 226112_at**	**SGCB**	**− 3.51**	**0.56**	**4q12**	**Cytoskeleton organization**
** **217901_at	DSG2	− 3.51	0.38	18q12.1	Cell adhesion
** **224955_at	TEAD1	− 3.50	0.44	11p15.2	Regulation of transcription
** 219855_at**	**NUDT11**	**− 3.50**	**0.63**	**Xp11.22**	**Vesicle trafficking, DNA repair**
** **201387_s_at	UCHL1	− 3.49	0.40	4p14	Protein deubiquitination /// negative regulation of MAP kinase activity
** 224650_at**	**MAL2**	**− 3.48**	**0.54**	**8q23**	**Protein transport**
** **222778_s_at	WHSC1	− 3.46	0.54	4p16.3	Chromatin modification /// regulation of transcription
** 59697_at**	**RAB15**	**− 3.45**	**0.66**	**14q23.3**	**Protein transport, signal transduction**
** **208180_s_at	HIST1H4H	− 3.39	0.57	6p21.3	Nucleosome assembly
** **202345_s_at	FABP5	− 3.35	0.51	8q21.13	Lipid metabolic process /// transport
** **224233_s_at	MSTO1 /// MSTO2P	− 3.34	0.72	1q22	Mitochondrion organization, protein polymerization
** 206218_at**	**MAGEB2**	**− 3.31**	**0.62**	**Xp21.3**	**Melanoma antigen family B2**
** **221606_s_at	HMGN5	− 3.28	0.59	Xq13.3	Regulation of transcription /// chromatin modification
** 206363_at**	**MAF**	**− 3.24**	**0.47**	**16q22-q23**	**Cytokine production /// regulation of transcription**
** 218597_s_at**	**CISD1**	**− 3.23**	**0.70**	**10q21.1**	**Regulation of cellular respiration**
** **219631_at	LRP12	− 3.21	0.62	8q22.2	Signal transduction
B					
** 242388_x_at**	**TAGAP**	**3.99**	**1.90**	**6q25.3**	**Signal transduction**
** 222790_s_at**	**RSBN1**	**3.81**	**1.51**	**1p13.2**	**Protein binding**
** **228007_at	C6orf204	3.75	1.63	6q22	—
** **225582_at	ITPRIP	3.60	1.58	10q25.1	—
** 227708_at**	**EEF1A1**	**3.53**	**1.73**	**6q14.1**	**Protein biosynthesis**
** **41220_at	SEPT9	3.50	1.61	17q25	Cell cycle
** 208873_s_at**	**REEP5**	**3.44**	**1.49**	**5q22-q23**	**Protein binding**

FDR, false discovery rate.

∗ Seventeen of these genes were retained in the final optimized predictor for early relapse (highlighted in bold).

Each gene's expression values were dichotomized, using the 75th percentile as a threshold between high and low expression for the up-regulated genes and the 25th percentile as a threshold for the down-regulated genes. These genes underwent further shrinkage and selection using the LASSO algorithm [24], yielding 17 genes with the strongest discriminative power for early relapse. The selected genes were fitted in a logistic regression model to generate an optimal gene-expression based predictor for likelihood of relapse within 1 year post-HDT (Table III).

**Table III.** The 17 selected genes were fitted in a logistic regression model to generate an optimal GEP-based predictor for likelihood of early relapse post-HDT, and the probability for each case could be calculated accordingly.

z = −1.2153 +210546_x_at(H)∗0.5535 + 223253_at(H)∗0.371+ 219895_at(H)∗1.588 + 207717_s_at(H)∗0.8155 + 204379_s_at(H)∗0.423 + 213194_at(H)∗0.835 + 226112_at(H)∗0.7029 + 219855_at(H)∗0.3427 + 224650_at(H)∗0.4121 + 59697_at(H)∗0.1446 + 206218_at(H)∗0.1698 + 206363_at (H)∗0.1265 + 218597_s_at(H)∗1.9405 − 242388_x_at(H)∗0.979 − 222790_s_at(H)∗0.8829 − 227708_at(H)∗0.8721 − 208873_s_at(H)∗1.3275
Probability (early relapse) = 1/1 + e^− z^

GEP, gene expression profiling; HDT, high-dose therapy.

The 17-gene signature (REL-17) had an AUC of 0.917 with an optimal sensitivity and specificity of 87% and 83%, respectively [Supplementary Figure 3(A) available online at http://informahealthcare.com/doi/abs/10.3109/10428194.2014.911863]. Its predictive capability was validated in an independent set of patients [AUC 0.804, Supplementary Figure 3(B) available online at http://informahealthcare.com/doi/abs/10.3109/10428194.2014.911863], which was a considerable improvement on the FISH-based predictor [AUC 0.69, Supplementary Figure 2(C) available online at http://informahealthcare.com/doi/abs/10.3109/10428194.2014.911863]. When tested in the validation set, adding t(4;14) and gain(1q) status to this GEP model did not statistically improve the predictive capability (likelihood-ratio test *p* = 0.21).

**Figure 2.**
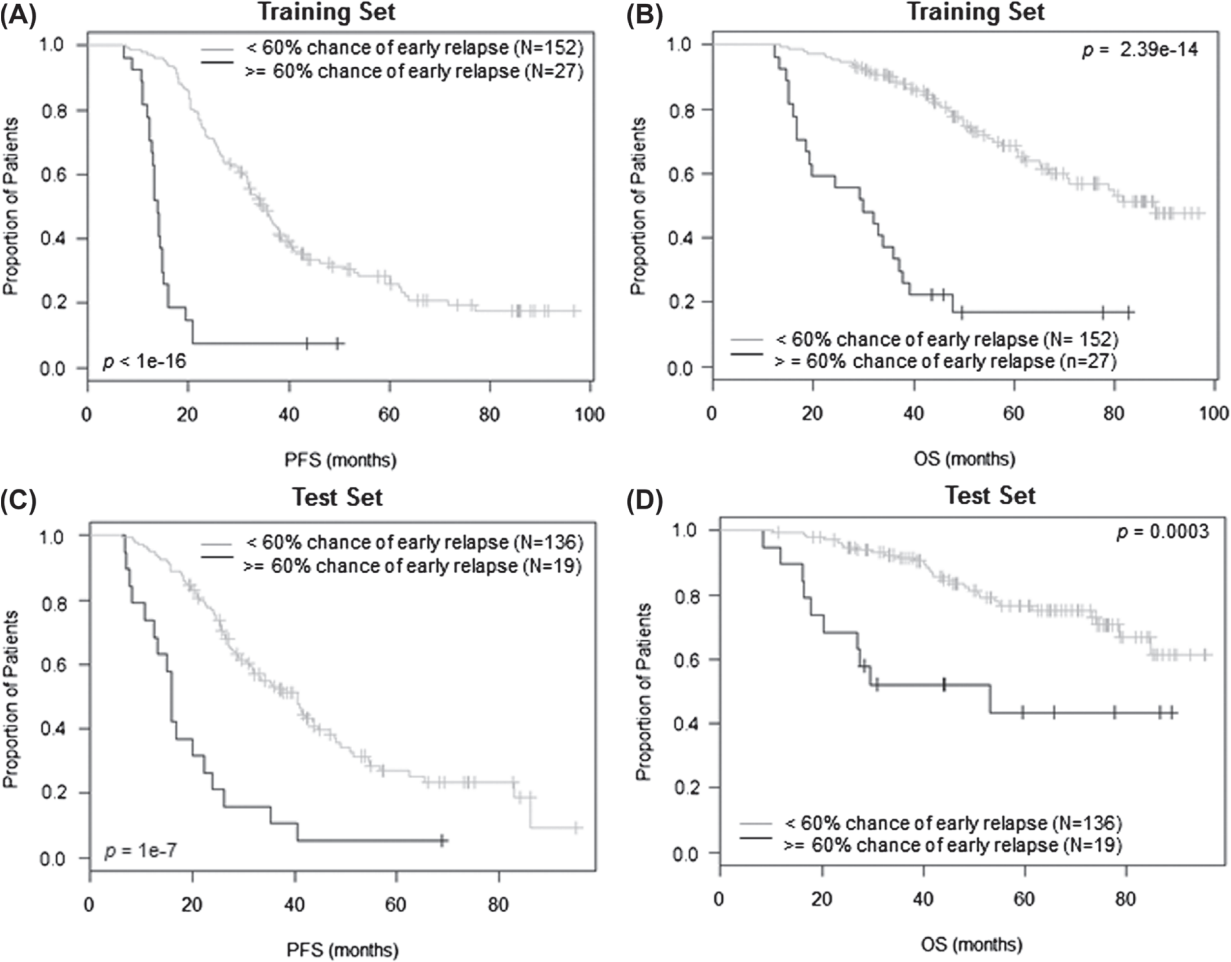
Effect of risk groups derived from the REL-17 signature on PFS and OS. In the training set, 15.1% cases being identified as having more than 60% chance to relapse within 1 year had significantly shorter PFS (A; median 13.8 vs. 34.8 months, *p* < 10^− 16^) and OS (B; median 29.9 vs. 88.1 months, *p* = 2.39 × 10^− 14^) in contrast to those at lower risk. Using the same criteria 12.3% patients being identified at high risk in the test set also had significantly shorter PFS (C; median 15.9 vs. 40.5 months, *p* = 10^− 7^) and OS (D; median 53 months vs. not reached, *p* = 0.0003) compared to rest of the cases.

Fifteen per cent of patients in the training set were identified as having a more than 60% chance of relapsing within 1 year, and this group had significantly worse PFS and OS [Figures 2(A) and 2(B)]. The significant associations with PFS and OS were validated in the test set [Figures 2(C) and 2(D)]. The risk groups derived from the REL-17 signature were also compared with those derived from the Erasmus University Medical Center (EMC)-92 signature [25] in multivariate analyses for their performance of predicting relapse within 1 year post-HDT, PFS and OS, respectively, in the independent test set. The results showed that the REL-17 signature performed best for predicting relapse within 1 year and PFS (Table IV), although was also associated with OS [Figure 2(D)]. We applied the REL-17 signature to a further independent dataset (GSE24080), either as a whole (*p*-value 2.03 × 10^− 9^ and 3.73 × 10^− 11^ for PFS and OS respectively) or in two subsets from separate trials (Supplementary Figure 4 available online at http://informahealthcare.com/doi/abs/10.3109/10428194.2014.911863), where its effects on PFS and OS were also validated.

**Table IV.** Risk groups derived from 17-gene early relapse signature (REL-17) were compared with those derived from EMC-92 signature in multivariate analyses for their performance predicting relapse within 1 year, PFS and OS in the test set (*n* = 155).

	OR/HR	95% CI	*p*-Value
Relapse < 1 year			
REL-17	9.69	2.99–31.38	0.0002
EMC-92	1.57	0.51–4.80	0.43
PFS			
REL-17	3.21	1.71–6.02	0.0003
EMC-92	1.26	0.72–2.21	0.41
OS			
REL-17	1.75	0.76–4.02	0.18
EMC-92	3.25	1.57–6.70	0.001

PFS, progression-free survival; OS, overall survival.

### Mechanisms of gene deregulation

We explored the possible mechanisms underlying the deregulation of the differentially expressed genes by carrying out integrative analyses of GEP, DNA methylation and SNP-mapping array data. Among the top 37 genes, expression levels of *MSTO1/MSTO2P* (on 1q22), *RSBN1* (on 1p13.2), *EEF1A1* (on 6q14.1) and *REEP5* (on 5q22) were positively correlated with copy number changes (Supplementary Figure 5 available online at http://informahealthcare.com/doi/abs/10.3109/10428194.2014.911863, *p* < 0.05).

We also looked at the correlation between the expression level of these top deregulated genes and the methylation status of their promoter, except for *NUDT11* and *MAL2*, for which there were no corresponding methylation probes. The methylation probes for these genes were all located in CpG dense areas and mapped to promoters or transcription start site, with their DNA methylation status following a binary distribution [Supplementary Figure 6(A) available online at http://informahealthcare.com/doi/abs/10.3109/10428194.2014.911863]. The expression levels of nine genes (*CTAG1, EPDR1, CTAG2, PKP2, NGFRAP1, LRIG1, NAP1L3, MAF, LRP12)* were statistically correlated with the methylation status (*p* < 0.05). Scatter plots for these genes show a typical “L” pattern, indicating that the hyper-methylation status prevents the genes being transcribed, which suggests that promoter DNA methylation is likely to make an important contribution to the transcription of these genes [Supplementary Figure 6(B) available online at http://informahealthcare.com/doi/abs/10.3109/10428194.2014.911863]. As *MAF* has been shown to be deregulated via t(14;16) and can also be induced via *WHSC1* translocation, the correlation for *MAF* was only analyzed in cases negative for both t(14;16) and t(4;14) by FISH, and a correlation with methylation status was seen. For the genes located on chromosome X, the correlations were also confirmed in each gender group separately (data not shown).

## Discussion

MM therapeutic schemes are changing rapidly with the constant introduction of new drugs. Although regimens containing novel agents may produce comparable response rates to those of HDT, prospective data based on a head-to-head comparison are limited, especially for the long-term effect. Therefore these agents are currently incorporated prior to and following the HDT rather than replacing the procedure as first-line regimen. The GEP signature in this study was developed and validated in a series of datasets using novel agents as induction and maintenance, which reflects the current treatment settings.

In this study we show that one of the most significant predictors for long-term survival following HDT-ASCT at presentation is the time to first relapse. Our analyses show a clear differential effect on post-relapse survival between patients who relapse within 1 year in contrast to those relapsing later. We used this observation as a tool to derive a GEP-based predictor for outcome that effectively identifies cases at high risk of early relapse. When tested in the training set, cases considered as being at high risk, having a > 60% probability of early relapse, were shown to have significantly shorter PFS and OS compared to the rest of the cases. The effects on both OS and PFS were validated in two independent datasets; interestingly, the prognostic merit of this predictor was also seen in the Total Therapy 3 (TT3) cohort, which comprises the most intensively treated cases so far (bortezomib/thalidomide-combined induction followed by double autograft with bortezomib/lenalidomide-combined maintenance) [26]. Therefore this signature seems to be applicable for all cases receiving HDT-ASCT regardless of the type of induction and maintenance therapy received.

We show that the presence of known FISH-based abnormalities, including adverse IgH translocations and gain(1q), are strongly associated with early relapse following HDT. However, in our analysis this FISH-based predictor only has a modest predictive performance, and therefore lacks the sensitivity and specificity to be used as a prognostic test. Furthermore these FISH abnormalities do not statistically improve the predictive capability of the REL-17 signature for high risk clinical behavior. This is consistent with observations from the EMC-92 [25] and University of Arkansas for Medical Sciences (UAMS) [14] signatures, suggesting that additional biological features, defined by the GEP, interact with behavior induced by the FISH variables to determine high risk behavior. As the number of genes comprising this signature is low and the risk score is calculated based on the binary expression status of each gene (high/low), it could be transformed into a reverse transcription-polymerase chain reaction (RT-PCR)-based test.

Among the 37 genes most differentially expressed between the two risk groups, the expressions of *FGFR3, WHSC1* and *DSG2* are known to be deregulated via t(4;14). The association of t(4;14) myeloma with aggressive relapse has been reported in a number of studies [27,28]. These genes, together with another five significantly deregulated genes, *NGFRAP1, NAP1L3, TEAD1, LRP12, AGAP1* (*CENTG2*), are among the overexpressed genes previously seen within this molecular subgroup [23]. *MAF*, another gene associated with early relapse, is a transcriptional activator of key target genes and is mainly deregulated via t(14;16) [29].

Gene enrichment analysis by chromosome location shows that the 207 deregulated genes (FDR < 0.05) were significantly overrepresented on chromosome 1 and X; notably the overrepresentation of genes on chromosome 1 was no longer seen among the top 37 deregulated genes (FDR < 0). These findings support the importance of genes in the 1q12-q23 region as outcome predictors, but also that there are other genes playing more important roles in determining high risk behavior, which are not able to be identified by the FISH approach. Despite the over-representation of genes from chromosome X, the risk of early relapse was not associated with gender (data not shown). Among the genes located on chromosome X, *CTAG1, CTAG2* and *MAGEB2* belong to the cancer testis gene family (CTAGs), which have been previously demonstrated to have prognostic value in patients with myeloma [30]. It is also noteworthy that the 37 top deregulated genes were enriched for the myeloma “stem cell” gene set [23], including *NUDT11, PKP2, ROBO1, AGAP1, NAP1L3* and *EPDR1*, indicating that “stemness” may play an important role in determining high risk behavior. It is interesting that one of these genes, *ROBO1*, together with another deregulated gene, *HMGN5,* is also present in the UAMS-70 gene signature [14].

Integrative analysis of GEP and SNP-mapping array data shows that only four of the 37 top differentially expressed genes are possibly deregulated via copy number variation. Two of them are located on 1q22 and 1p13.2, respectively, which may reflect the association of gain(1q) and del(1p) with inferior outcome. The majority of the top differentially expressed genes do not appear to be deregulated via mechanisms which could be detected by FISH, such as translocations or gains/losses. The exploratory analysis integrating GEP and DNA-methylation profiling shows that nine of these genes were statistically associated with the DNA methylation level at the promoter, suggesting an epigenetic mechanism being involved in their transcription, among which CTAG genes have been previously shown to be silenced by DNA methylation during normal cellular differentiation [31]. We also found significant evidence showing that three “stem cell” genes, *PKP2, NAP1L3* and *EPDR1*, might be modulated via DNA methylation. Although *MAF* is normally deregulated via t(14;16) and t(4;14) in MM, there are still cases that express this gene while lacking these translocations, consistent with additional unknown mechanisms driving its transcription. In this analysis the association between *MAF* expression and the promoter methylation status suggests a possible epigenetic mechanism in its transcription, as is frequently seen in diffuse large B-cell lymphomas [32].

In conclusion, in this work we have developed a GEP- based predictor for high risk myeloma treated with HDT-ASCT. The signature is biologically relevant and can identify individuals, who constitute up to 20% of newly diagnosed patients with myeloma, whose remission is not sustainable, with a high risk of relapsing within 1 year post-HDT. Patients identified via such an approach could have their treatment modified to improve outcomes. The future development of predictive signatures is likely to focus on the use of biologically relevant genes which are deregulated via specific mechanisms.

## Supplementary material available online

Tables and figures showing further results
